# Preparation of 3D Printed Polylactic Acid/Bacterial Cellulose Composite Scaffold for Tissue Engineering Applications

**DOI:** 10.3390/polym14214756

**Published:** 2022-11-06

**Authors:** Yadong Wu, Yunfeng Wang, Fang Wang, Yudong Huang, Jinmei He

**Affiliations:** 1 MIIT Key Laboratory of Critical Materials Technology for New Energy Conversion and Storage, School of Chemistry and Chemical Engineering, Harbin Institute of Technology, Harbin 150001, China; 2 Laboratory for Space Environment and Physical Sciences, Harbin Institute of Technology, Harbin 150001, China

**Keywords:** 3D-printed, polylactic acid, bacterial cellulose, composite scaffold materials, biocompatibility

## Abstract

Bacterial cellulose (BC) has become a universal biomaterial owing to its intrinsic properties. BC fibers are composed of microfibers with a diameter of 3–4 nm into fiber bundles with a thickness of 40–60 nm, and interweave with each other to form a well-developed ultra-fine network structure. Polylactic acid (PLA) has good mechanical properties and excellent biocompatibility and biodegradability. Therefore, PLA has been widely applied in tissue engineering. Addressed herein is a novel type of PLA/BC (PLA/BC) composite scaffold prepared by 3D printing (3D), 3D modeling of the required porous membrane material support established in the computer, and decomposition of the model into 5 layer 20 μM sheets. The range of PLA loadings assessed in this work was 1.0 wt.%, 1.5 wt.%, and 2.0 wt.%, and its physicochemical properties and biological properties were characterized and evaluated. Tensile strength of PLA/BC composite scaffolds increased to 66.49 MPa compared to that of a pure BC film (25.61 MPa). Hydrophilicity was tunable with the amount of added PLA. In this paper, the effects of 3D round hole and stripe surface topology on cell growth behavior were characterized. Schwann cells (SCs) adhered to the surface of the 3D composite membrane successfully, and their proliferation rate on the surface of the regular circular pore and stripe structure was better than that of the smooth surface. Erythrocyte fixation and platelet adhesion experiments showed that the 3D composite scaffold had excellent blood compatibility. Further degradation studies showed that loose structures appeared after 1 week, and structural defects began after 3 weeks. The in vitro degradation results showed that the degradation rate of the BC membrane in simulated body fluid after 6 weeks was 14.38%, while the degradation rate of the PLA/BC composite scaffold was 18.75%.

## 1. Introduction

Being a research hotspot today, tissue engineering is widely used in maintenance, repair, replacement and reconstruction [1,2,3,4,5]. Traditional scaffolds and engineering methods limit their ability to produce tissue structures with accurate bionic characteristics. 3D bioprinting technology provides unprecedented versatility in the field of tissue engineering and realizes the composition and the precise control of spatial distribution. Their construction accuracy enables the preparation of tissue engineering scaffolds with fine shape and structure. Even if we join the timing dimension, we can continue to expand the use of 4D printing technology to enhance the performance of our products [6]. The rapidly evolving of 3D and 4D printing technologies are making suitable appropriate materials become the key momentous elements in the field of biomaterials [7,8]. It is encouraging that the related work has been carried out and has achieved impressive results [9,10,11].

PLA is an aliphatic polyester separated from a wide range of sources, such as corn, wheat, and sugar beet, it is a fully biodegradable thermoplastic polyester that has the potential to become a substitute for traditional petroleum-based polymers [12,13,14]. Owing to its specifications, it is widely used in numerous applications [15,16,17,18]. Due to its excellent degradability and biocompatibility, PLA is often used in the biomedical field [19,20]. However, the Suboptimal crystallinity of PLA and the obvious friability of printed products limit its extensive application prospects [12]. 

As the most abundant biopolymer resource, plant cellulose is a kind of biodegradable and renewable polysaccharide derived from plants such as cotton and wood [21,22]. Nowadays, multitudinous studies to research the synergism between nanocellulose and different matrix polymers have been carried out [23,24,25,26,27,28]. Nanoscale cellulose fiber has been used in 3D printing to enhance the mechanical properties of PLA and other polymers [29,30,31,32]. It is striking that BC has a similar physical and chemical structure with natural plant fiber, but no complex components, such as hemicelluloses, lignin, pectin, and cell walls. BC fibers are composed of microfibers with a diameter of 3–4 nm into fiber bundles with a thickness of 40–60 nm, and interweave with each other to form a well-developed ultra-fine network structure. A variety of ways to culture BC have been used, such as th microbial system and cell-free enzyme systems [33,34]. Due to its unique three-dimensional interpenetrating network structure, BC has the advantages of biodegradability, sterilization, and excellent biocompatibility in the application of biomaterials. Studies have shown that micro and nanoscale scaffolds are more suitable for cell growth and the formation of new tissues [35,36]. Therefore, BC has become a research focus in the field of new tissue engineering biomaterials in recent years [37,38,39,40].

It is distressing that due to the insufficient cellulase in the human environment and a weak alkaline environment in vivo, the degradation process of BC is hindered and results in a the slow degradation process. Therefore, we used PLA, which can be degraded into small molecules of lactic acid in the internal environment and absorbed by the human body without causing toxicity or inflammatory reaction to improve the degradation ability of BC. In this study, PLA/BC composite scaffolds were prepared by 3D-printing technology, which has been demonstrated as an effective strategy to enhance the performance of the scaffold. The effect of PLA addition on the physicochemical and biological properties of PLA/BC composite scaffold was studied by adopting FTIR, XRD, tensile strength measurements, and biocompatibility evaluation. 

## 2. Materials and Methods

### 2.1. Materials

White sugar and black tea were obtained from Rui Fu food Co., Ltd., in Tainan, Taiwan. L-PLA, sodium hydroxide (NaOH) and Potassium hydroxide (KOH) were purchased from Alfa-Aesar Co., Ltd., inTewksbury, MA, USA. DEME high glucose medium and fetal calf serum were purchased from Gibco, USA. All chemicals were used as received without further purification. 

### 2.2. Preparation of BC Membrane

In this study, BC was cultivated by the static culture method. White granulated sugar (20 g), water (200 mL), and tea leaves (2–3 g) were placed in a beaker and stirred (200 rpm/min) for about 2 h to dissolve homogeneously. The solution was subsequently boiled to wholly remove hetero bacteria. After the medium cooled down to room temperature, pellicle film (8 mm) was added with the appropriate amount of bacterial fluid, then the beaker was placed in a dark and cool place to ensure good ventilation, while keeping dust from entering with gauze. On Day 7, translucent film (2 mm) formed on the surface of the medium liquid as BC gel.

After pH detection, the BC gel above was acidic and contained medium residue, which needed to be purified. The BC gel was rinsed with plenty of distilled water to remove residual medium. Then, it was soaked in a 8.0 wt.% NaOH solution and stirred at room temperature for 4 h to remove the bacteria and medium residue. It was continuously rinsed with copious amounts of distilled water to remove residual NaOH until it was neutral, and the purification was completed. The BC gel was cut into pieces and placed in a beating machine, and an appropriate amount of distilled water was added to make a BC slurry with a BC weight percentage of 33%, which was stored at 4 °C. Finally, 25 g of this slurry were removed and collected by suction filtration to form membranes at room temperature. Each BC membrane was weighed at 80 g for pending use.

### 2.3. Preparation of 3D-Printed PLA/BC Composite Scaffolds

In this paper, three models are established, which are two kinds of circular-hole-distributed PLA and square-lattice-distributed PLA with different sizes, which were called PLA/BC-1, PLA/BC-2, and PLA/BC-3 composite scaffolds, respectively. The specific operation steps were divided into the following three parts:

(1) Modeling: established the 3D model of the required porous membrane material support in the computer, and decomposed the model into 5 layer 20 μM sheets;
(2) Feeding: added PLA powder for 3D printing into the barrel of the 3D printer;
(3) Printing: PLA was printed onto the BC membrane material placed on the three-dimensional fluctuation platform by layer printing. Among them, the mass of the PLA used in PLA/BC-1, PLA/BC-2, and PLA/BC-3 were 80 mg, 120 mg, 160 mg, respectively.

**Figure 1:**
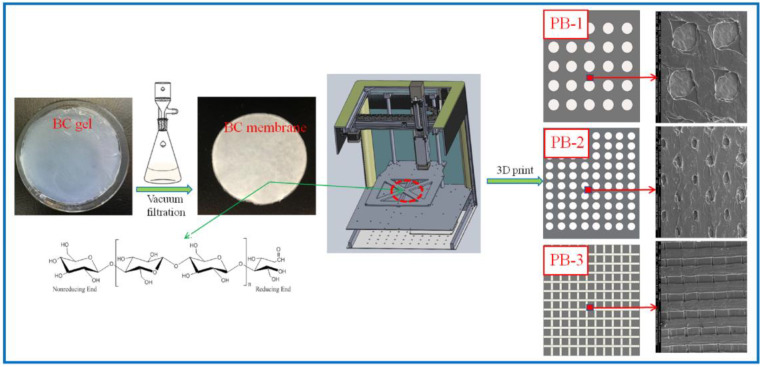
Schematic diagram of the preparation process of PLA/BC composite scaffolds.

The percentage of PLA in the three composite scaffolds was calculated to be 1 wt.%, 1.5 wt.%, and 2 wt.%. The schematic diagram of the preparation process of the PLA/BC composite scaffolds is exhibited in Figure 1; moreover, the structural formula of the BC is also shown in Figure 1.

### 2.4. Characterization and Performances Study of PLA/BC Composite Scaffolds

The surface morphology of the BC membrane and the composite scaffold hybrid membranes were analyzed using scanning electron microscopy (SEM, Apreo S HiVoc) with an accelerating voltage of 5–20 kV. ATR-FTIR studies were performed to investigate the functional groups of scaffold surfaces (Scan range of wavenumber: 400–4000 cm^−1^). XRD measurement was carried out by a D/man-rBX X-ray generator operated at 30 mA and 40 kV to study the crystalline structure. Thermo-gravimetric analysis of the scaffolds was employed by TGA (TA Q500), using a heating rate of 10 °C/min from 30 to 800 °C in the atmosphere of nitrogen. Surface hydrophilic behavior of the composite scaffolds was evaluated by measuring the static water contact angle. The measurement was conducted on a SL200KB drop shape analysis system (Shanghai, China). Double-distilled water was used as the probe liquid. The measurement of a given contact angle was carried out for at least five times. The mechanical strength of the synthesized scaffold was tested by a Cmt8102 electric universal testing machine with a tensile speed at 10 mm/min at room temperature. The tensile strength measurements were repeated five times for each specimen, and the average tensile strength was recorded.

### 2.5. Biological Properties Evaluation of PLA/BC Composite Scaffolds

Degradation properties in vivo: The BC membranes and PLA/BC composite membranes were embedded into the inner thighs of New Zealand white rabbits, and at 1, 2, and 3 weeks of embedding, the newly generated tissues were removed together with the materials, rinsed with PBS, and then fixed by immersion in 4% paraformaldehyde solution for 24 h. The membranes were treated with graded alcohols and placed in xylene solution to make them transparent, then immersed in wax cylinders for paraffin embedding. Serial sections of the transverse and longitudinal sections were performed, placed in an oven (75 °C) overnight, and finally were H & E stained before light microscopy observation and image acquisition.

Red blood cell fixation experiments: The disc-shaped membrane was placed inside the wells of a 24 well plate, and the materials were sterilized with 75% alcohol for 30 min each, 3 times, followed by replacing the residual ethanol with sterile PBS buffer for 10 min each, 3 times. The membranes were labeled with their corresponding names, placed into the well plate, and 100 μL of whole blood were added, ensuring that the sample was sufficiently infiltrated. The well plate was then into the 37 °C incubator to make blood contact with the material for 5 min. After that, sterile PBS was added to each well and rinsed three times, then fixed by adding 2.5% glutaraldehyde for 2 h, and then dehydrated by alcohol gradients, that is, dehydrated by 30%, 50%, 70%, 90%, 100% alcohol, for 2 times for 20 min each, after which the samples were transferred to a freeze-drying box to dry completely. After the sample surfaces were gold sprayed, the micro morphology of the samples was observed by scanning electron microscopy.

Platelet adhesion experiments: Fresh blood was collected in centrifuge tubes and centrifuged at 1000 r/min for 5 min to obtain the supernatant, which was defined as blood platelet rich plasma (PRP). The remaining blood was centrifuged at 3000 r/min for 10 min, and the supernatant, i.e., platelet poor plasma (PPP), was collected to adjust the PrP with PPP, so that the concentration of platelets in the PRP was 3 × 10^8^/L. The disc-shaped membrane was placed inside the wells of a 24 well plate, and the materials were sterilized with 75% alcohol for 30 min each, 3 times, followed by replacing the residual ethanol with sterile PBS buffer for 10 min each, 3 times, so that the materials were thoroughly moistened and blotted with PBS. The materials were labeled with their corresponding names, placed into the well plate, and then the platelets were added to the PBS buffer at a volume ratio of 1:20, and finally the well plate was placed into the 37 °C incubator to react for 1 h. After that, sterile PBS was added to each well rinsed and three times, then fixed by adding 2.5% glutaraldehyde for 2 h, and dehydrated by alcohol gradients, that is, dehydrated by 30%, 50%, 70%, 90%, 100% alcohol, twice for 20 min each time. The cell samples were transferred to the freeze-drying box to dry completely. After the sample surfaces were gold sprayed, the micro morphology of the samples was observed by scanning electron microscopy.

Cytotoxicity experiment: Human umbilical vein vascular epithelial cells (HUVECs) and mouse embryonic fibroblasts (3T3) were used for toxicity evaluation. The frozen cells were resuspended and placed in a culture flask and cultured in high glucose DMEM medium containing 10% fetal bovine serum and 1% double antibody (1640 medium was used for HUVECs). After the cells grew to 80% of the bottom area of the flask, the cells were digested with trypsin and terminated by adding a DMEM/1640 medium containing 10% fetal bovine serum. After gently pipetting with a pipette, the cells were centrifuged at 1500 rpm for 3 min in a 15 mL centrifuge tube, and the supernatant was discarded. The appropriate amount of culture solution was added again by pipetting to make the cells into a homogeneous suspension. After counting with a cell counting plate, the cell suspensions were dispensed into the desired cell concentration and then seeded onto a 96 well plate at 4000/100 μL of the culture solution. After the cells were grown for 24 h inside the plates, the medium was discarded and 100 μL was added to each well of the immersion extract, which were divided into the experimental group (composite scaffold), the control group (BC) and the blank group (fresh DMEM/1640 medium). The 96 well plates were placed in a 37 °C carbon dioxide incubator and incubated for 48 h to perform MTT assay. Specific procedures: the extract was discarded and protected from light, 20 μL was added to each well of a 5 mg/mL MTT dye and incubated for 4 h in an incubator. The MTT was aspirated with 150 μL of DMSO and shaken for 10 min to dissolve the blue-purple crystals. For cell-free wells, an equal amount of DMSO was added as a control. The absorbance of each well at 490 nm, the OD 490 nm value, was measured by a microplate reader.

Schwann Cell adhesion experiment: The disc-shaped membrane was placed in the wells of a 24 well plate, and the material was sterilized with 75% alcohol for 30 min each, three times, followed by replacing the residual ethanol with sterile PBS buffer for 10 min each, three times, which was washed with PBS and given fresh medium to wet the material before cell seeding. To select the desired well plate, the cells were labeled with their corresponding names, placed into the well plate, 1 mL of DMEM medium was added to each well, placed in a cell culture incubator for 2 h and then aspirated, and the medium was discarded. After SCs were digested with 0.25% trypsin, the digestion was terminated with DMEM medium containing 10% fetal bovine serum; the supernatant was discarded by centrifugation (1500 RMP, 3 min) in a centrifuge tube, and the cells were resuspended with fresh medium. Cells were seeded onto scaffolds in 24 well plates at 50,000 cells in each well for 15–20 min, and 1 mL of DMEM containing 10% fetal bovine serum and 1% double antibody was added to each well. The cell culture plates were placed in a CO_2_ incubator at 37 °C and replaced with fresh culture solution every 3 days. After 24 h of cell culturing, the scaffolds seeded with cells were washed twice with sterile PBS, and 4% paraformaldehyde was added to fix the cell/scaffold composites for 15 min. They were then dehydrated with alcohol gradients, i.e., 30%, 50%, 70%, 90%, and 100% alcohol, twice for 20 min each, respectively, and the cell samples were transferred to the freeze-drying box to dry completely. After the sample surfaces were gold sprayed, the micro morphology of the samples was observed by scanning electron microscopy.

SCs immunological staining: After 24 h of seeding, the SCs were removed from the incubator, the culture medium was aspirated and discarded, and a group (six well) of cells were selected to add fresh DMEM medium, BC membrane leaching solution, and PLA/BC membrane leaching solution. After continuing the incubation for 24 h, the medium was aspirated and the cells were rinsed three times with PBS buffer at 37 °C and placed on a shaker for 5 min each time. They were then fixed with 4% paraformaldehyde solution for 30 min. After being well fixed, they were rinsed with PBS buffer 3 times, and each time placed on a shaker for 5 min. Cells were permeabilized by adding 1 mL of 0.1 wt.% Triton X-100 (Permeabilizer) in the PBS for 15 min, rinsed 3 times with PBS, and placed on a shaker for 5 min each time. To further counterstain the nuclei, 80 μ L of DAPI staining solution were incubated for 10 min in the dark at room temperature, rinsed again three times with PBS, and placed on a shaker for 5 min each time. Finally, the cells were observed under a fluorescence microscope and photographed.

SCs proliferation experiments: The circular membrane was placed inside the wells of a 24 well plate, and the materials were sterilized with 75% alcohol for 30 min each, three times, followed by replacing the residual ethanol with sterile PBS buffer for 10 min each, three times, and cell seeding was performed after the fresh medium was added to wet the materials by blotting the PBS. After SCs were digested with 0.25%-EDTA, the digestion was stopped with a DMEM medium containing 10% fetal bovine serum. The supernatant was discarded after being transferred to a centrifuge tube for centrifugation (1500 rmp, 3 min), and the cells were resuspended with fresh medium. Cells were seeded onto scaffolds in 24 well plates, 10,000 per well, containing 1 mL DMEM medium with 10% fetal bovine serum and 1% double antibody. The plates were placed in a CO_2_ incubator at 37 °C, and the medium was changed every three days. On days 1, 3, 5, and 7 of cell culturing, the culture plates were removed for proliferation rate assay. The specific procedures were as follows: The culture medium was discarded by suction, protected from light, added with 100 μL MTT dye solution (5 mg/mL), then incubated in a CO_2_ incubator at 37 °C for 4 h. The MTT solution was aspirated with 600 μL of DMSO and shaken for 10 min. After the Blue Violet crystals had been dissolved, they were pipetted in quantities of 150 μL into a 96 well plate. For cell-free wells, an equal amount of DMSO was added as a control. The absorbance of each well at 490 nm, the OD 490 nm value, was measured by a microplate reader. Relative Growth Rate (RGR) was calculated as follows: RGR = (value of OD 490 nm in the experimental group/value of OD 490 nm in the blank group) × 100%

## 3. Results and Discussion

### 3.1. Structural Morphology Observation

**Figure 2:**
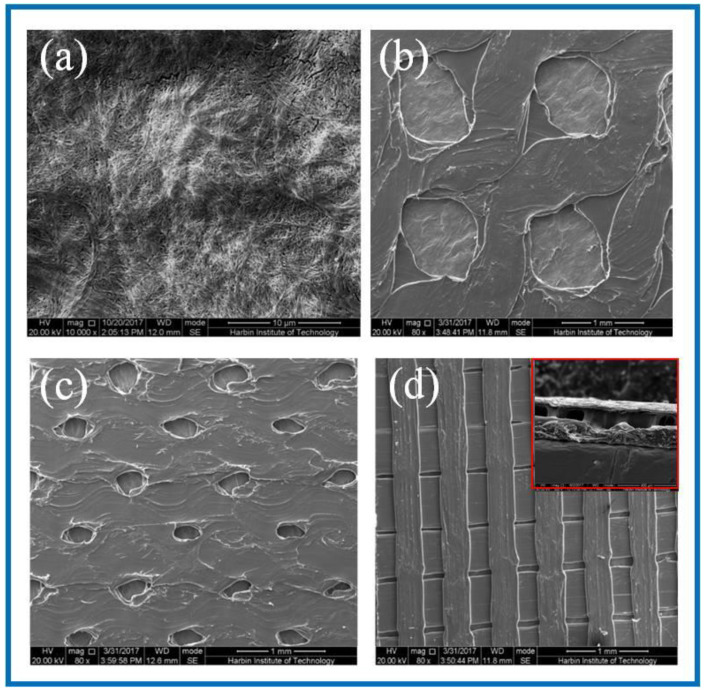
SEM images of BC membrane and 3D-printed PLA/BC composite films with three different surface microstructures at the same magnification of (**a**) pure BC membrane; (**b**) PLA/BC-1; (**c**) PLA/BC-2; (**d**) PLA/BC-3 (inset: cross section of PLA/BC-3).

SEM was used to observe the surface microstructure of the PLA/BC composite scaffold prepared by 3D printing. The surfaces morphology is shown in Figure 2.

As can be seen from Figure 2a, the dense structure of the BC was composed of ultra-fine bundle fibers. High density micro fibers were intertwined and wound to form a three-dimensional network structure with many pores, and the pores were evenly distributed. According to the measurement and analysis from Image J software (Version: 1.8.0, National Institutes of Health, Bethesda, USA), the diameter of the nanofibers on the BC surface ranged from 23–68 nm, with an average diameter of 41 nm, belonging to nanofiber structure. It can be seen from Figure 2b that the PLA printed on the surface of the PLA/BC-1 group of samples was in the shape of a circular hole, and the hole diameter was 950 ± 40 μm × 960 ± 30 μm. The distribution of circular holes was uniform, and the pore size was basically the same. As shown in Figure 2c, the PLA on the surface of the PLA/BC-2 presented in the shape of circular holes with pore diameters of about 250 ± 50 μm × 350 ± 50 μm. The distribution of circular pores was uniform, while the pore size was slightly different. As shown in Figure 2d, the PLA on the surface of the PLA/BC-3 group samples was in the shape of double-layer stripes, and the width of the bottom stripe was 460 ± 40 μm. The interval was about 58 μm. The width of surface stripe was 260 ± 40 μm. The interval was about 280 μm. The stripes were evenly distributed, and the surface presented a regular strip structure. An SEM image of the cross-section of the PLA/BC-3 was recorded as the inset. Comparatively speaking, both the PLA laminate and the BC substrate could be clearly observed, proving the successful formation of the PLA layer on top of the BC membrane.

### 3.2. Characterization and Performance Study of PLA/BC Scaffolds

#### 3.2.1. IR Studies of PLA/BC Composite Scaffolds

**Figure 3:**
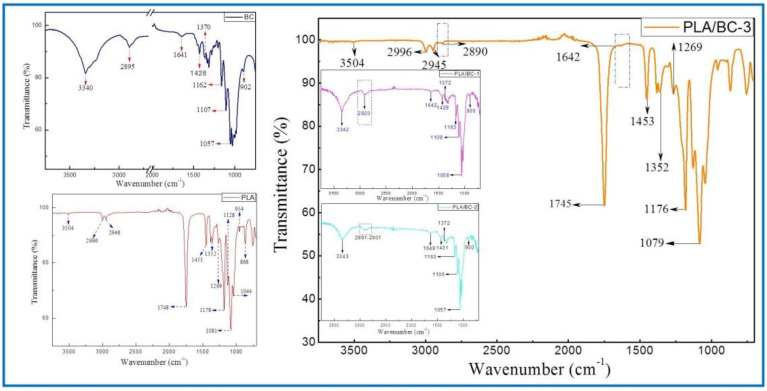
FTIR spectra of BC (left) and 3D−printed PLA/BC scaffolds (right).

The structures of the BC membrane, pure PLA and PLA/BC composite scaffolds prepared by 3D printing were analyzed by infrared spectroscopy, and the results are shown in Figure 3.

The ATR-FTIR spectra of the BC demonstrated typical spectral peaks. The -OH stretching and bending was located at around 3340 cm^−1^, -CH_2_ bending vibrations were at 1428 cm^−1^, and the C-H stretching and bending appear at 2895 cm^−1^ and 1370 cm^−1^. The bands at 1170–1050 cm^−1^ (1162 cm^−1^, 1107 cm^−1^ and 1057 cm^−1^) were ascribed to the vibrations of C-O-C bonds of the glycosidic bridges. The absorption bands at 902 cm^−1^ were characteristic of β-linked glucose-based polymers. Finally, the peak at 1641 cm^−1^ was assigned to the H-O-H in-plane deformation vibration of absorbed water by the BC. All the characteristic peaks corresponded to those of the BC, which indicated that BC was successfully fermented and cultured in this subject, and its structure was in accordance with the literature [41].

The spectrum of the PLA demonstrated the characteristic peaks corresponding to the PLA molecular structure. The stretching and bending peaks of the –OH were apparent at 3504 cm^−1^ and 1044 cm^−1^, respectively. The peaks at 2996, 2946, 1453, and 1352 cm^−1^ were the asymmetric stretching, symmetric stretching, symmetric bending, and asymmetric bending of −CH-, respectively. The stretching and bending peaks of the C=O appeared at 1748 cm^−1^ and 1269 cm^−1^, respectively. The peaks at 1178, 1128, and 1081 cm^−1^ were attributed to the C–O stretching. In addition, the peaks at 954 cm^−1^ and 868 cm^−1^ corresponded to the stretching of the C–C single bond [42].

Due to the high cellulose content, many similarities with the BC spectrum could be observed in the infrared curves of the PLA/BC-1 and PLA/BC-2 composite materials. The difference was that, with the addition of PLA, the band at 2895 cm^−1^ became wider than that of pure the BC, and shifted to a higher wavenumber segment. Furthermore, as the surface of the BC was almost completely covered by PLA, as shown in Figure 2d, the ATR-FTIR spectra of the PLA/BC-3 were basically consistent to PLA. By comparing the curves, the peaks at 2890 cm^−1^ and 1642 cm^−1^ appeared in the FTIR of the BC. In summary, no new absorption peaks appeared in the spectra. This can be explained by the fact that the PLA was just combined with the BC by physical interactions, without forming new functional groups.

#### 3.2.2. XRD Analysis of PLA/BC Composite Scaffolds

**Figure 4:**
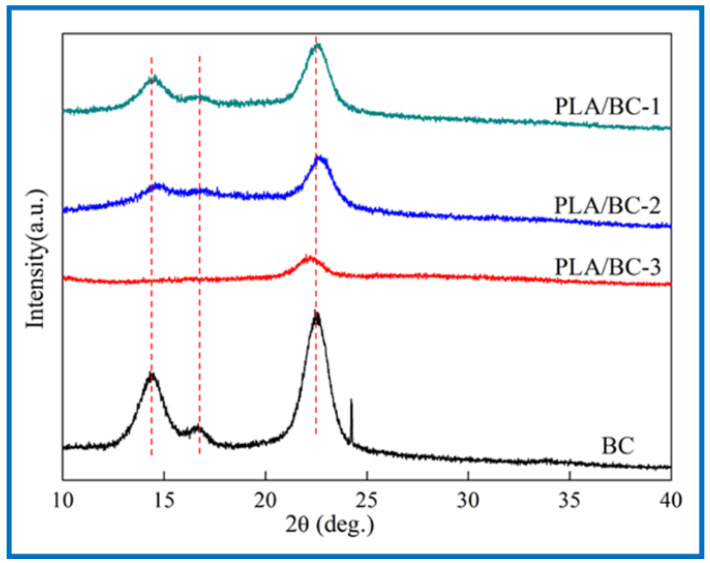
XRD spectra of 3D-printed PLA/BC scaffolds with three different surface microstructures.

Crystallinity is an essential property for a polymer membrane, as a change in crystallinity of the polymer can affect its physical properties. X-ray diffraction is mainly used to confirm the crystal form of the BC. The Bragg angle can be obtained from the data of diffraction peaks to calculate the corresponding parameters of cellulose crystals [43]. In 1984, Atalla et al. proved that two homogeneous and heteromorphic crystals could exist in natural cellulose crystals at the same time [44,45], which were named as cellulose I α and cellulose I β. XRD spectrums of the BC and PLA/BC scaffolds are shown as Figure 4.

The three characteristic peaks of the BC can be clearly observed from Figure 4. The Bragg angles at 14.5°, 16.8°, and 22.6° corresponded to (1ī0), (110), and (200) crystal planes, respectively [46], which are typical cellulose I crystals consistent with the XRD diffraction peaks of BC in relevant literature [47]. From the data of the obtained X-ray diffraction patterns, the crystallinity index (CrI) can be calculated according to Equation (1) [48]:

CrI = (*I_(200)_* − *I_(am)_*)/*I_(200)_* (1)

where *I_(200_*_)_, *I_(am)_* represent the diffraction intensity of (200) crystal planes at 22.6° and the minimum diffraction intensity between (110) and (200) crystal planes, respectively. The CrI of the BC was about 81%, and the results of the PLA/BC-1, PLA/BC-2, and PLA/BC-3 were approximately 67%, 63%, and 51%, respectively. In the process of compounding with the PLA, with the increase in PLA, the crystallization peak at 14.5° disappeared, and other peaks gradually became gentle. The diffraction curve of the amorphous system indicated that the PLA and BC were successfully compounded in the process of 3D printing, and the crystallinity of the composite was reduced by the influence of low crystallinity PLA.

#### 3.2.3. Thermal Properties of PLA/BC Composite Scaffolds

**Figure 5:**
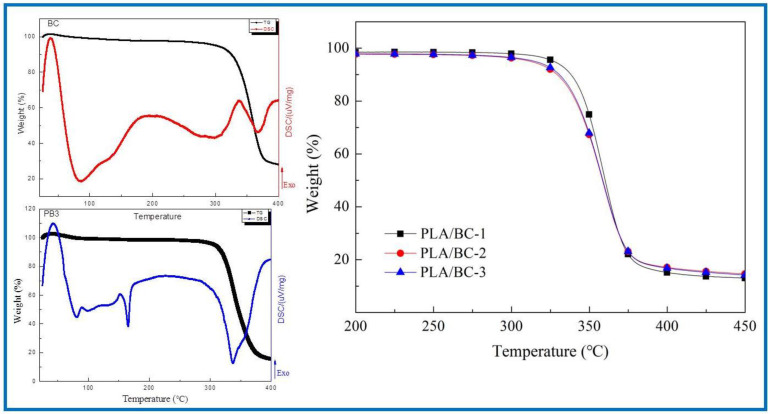
Analysis of thermal properties of BC membrane and PLA/BC composite scaffolds.

In the field of tissue engineering, the thermal properties of scaffolds play an important role. Firstly, the scaffold must have good thermal stability to ensure its stable existence in the human environment to provide the adhesion sites of cells and tissues. Secondly, in the process of support processing, the support material must resist a certain processing temperature without damage to its structure. In addition, the sterilization process before the use of the stent involves high-temperature sterilization, which also requires the material to have good thermal stability. In conclusion, it is essential to characterize the thermal properties of the scaffold. In this subject, the thermogravimetric analysis method was used to analyze the thermal properties of several materials and compare the thermal properties of the three PLA/BC composite films with different surface microstructures. First, the thermal properties of the BC membranes were characterized by TG-DSC. The thermogravimetric curve of the PLA/BC composite scaffolds are shown in Figure 5. Further, the TG-DSC of the PLA/BC-3 was carried out to compare to that of the pristine BC membrane.

As shown in Figure 5, the BC membrane began to decompose at around 317 °C, attributing to its high crystallinity and high purity. The thermal decomposition temperatures of three different 3D-printed PLA/BC composite scaffolds were 328 °C, 315 °C and 307 °C respectively, showing good thermal stability. With the addition of the PLA, a new endothermic peak appeared in the DSC curve of the PLA/BC at 165 °C, which suggested a decline in the crystallization of the material and was in accordance with the previous XRD results. Considering that the high-temperature sterilization environment is generally below 120 °C, the thermal properties of this scaffold were fully satisfied.

#### 3.2.4. Porosity Test of PLA/BC Composite Scaffolds

For porous scaffolds, ideal porosity is not only conducive to the smooth infiltration of nutrient solution and cell suspension into scaffolds to provide sufficient nutrient supply, but it also provides a good channel for the discharge of metabolites. BC has good porosity, in the process of compounding with PLA, it is hoped that the compounding process will improve its mechanical properties and maintain its high porosity structure, even though PLA itself does not have high porosity. Therefore, by giving its surface microstructure with the help of the 3D printing process, its porosity structure could be improved to a certain extent. Table 1 shows the porosity of the BC and PLA/BC membranes. Statistical comparisons were performed using SPSS 16.0 software, and difference were considered significant for *p* < 0.05.

It can be found that the porosity of the PLA/BC composite materials were reduced because the 3D-printed membrane layers had porous structures with large pore diameters and large spacing between micropores, which were consistent with the structure of the sample surfaces observed in the SEM diagram. However, the porosity remained above 80%. Therefore, the BC membrane and the PLA/BC composite membrane could be used in tissue engineering.

#### 3.2.5. Effect of PLA Loading on Hydrophilicity of the Composite Scaffolds

**Figure 6:**
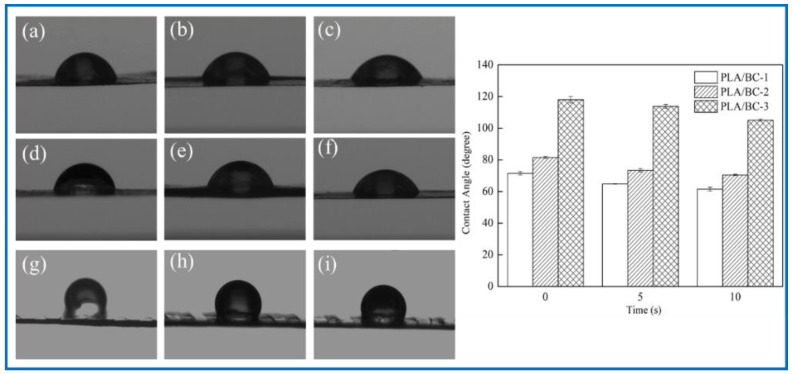
Water contact angle diagram under different contact time: (**a**–**c**): 0 s, 5 s, 10 s of PLA/BC-1; (**d**–**f**) 0 s, 5 s, 10 s of PLA/BC-1; (**g**–**i**): 0 s, 5 s, 10 s of PLA/BC-3.

To explore the effect of the introduction of the PLA on the hydrophilicity of the PLA/BC composite scaffolds, the water contact angle of three 3D-printed PLA/BC composite membranes with different surface microstructures were tested. The test results are shown in Figure 6.

As shown in Figure 6, the water contact angle of the PLA/BC-1 was 71.48 ± 1.03°, and gradually changed to 64.80 ± 0.15° after 5 s, then decreased to 61.51 ± 1.28° after 10 s. The initial contact angle of the PLA/BC-2 was 81.52 ± 0.58°, and gradually changed to 73.44 ± 1.02° after 5 s, and decreased to 70.52 ± 0.46° after 10 s. The initial contact angle of the PLA/BC-3 was 117.99 ± 2.03°, and gradually changed to 113.75 ± 1.32° after 5 s, and then decreased to 105.11 ± 0.7° after 10 s. Comparing the data of the three membranes, it was found that the water contact angles of the PLA/BC-1 and PLA/BC-2 composite membranes were lower than 90° which showed its hydrophilic, while the water contact angles of the PLA/BC-3 were above 90°, which had been transformed into hydrophobic membranes. The reason for the phenomenon above was that abundant hydrophilic hydroxyl groups existed on the surface of the BC and endowed it with good hydrophilicity. The ester group contained in PLA was a hydrophobic group, which made its hydrophilicity poor. The introduction of the PLA reduced the hydrophilicity of the PLA/BC composite membranes, and its hydrophilicity decreased with the increase in PLA content. Therefore, it was necessary to control the appropriate ratio of BC to PLA to obtain PLA/BC composite membranes with ideal hydrophilicity. In addition, another important reason for the change in the contact angle was the varieties of structure of the PLA film. It was preliminarily speculated that the diameter of the PLA circular hole on the surface of the PLA/BC-1 would large enough to expose BC film. In the water contact angle test, contact area of the 10 μL water droplet was smaller than the diameter of the circular hole, so the contact position was probably the pure BC membrane interface or the interface between the PLA and BC, making the value of contact angle relatively small. The circular pore diameter of the PLA film in the PLA/BC-2 was too small to accommodate the 10 μL water drop, so the water drop contacted the PLA mainly, and its contact angle increased. The PLA/BC-3 was a double-layer stripe structure. Its BC surface was completely covered by PLA, as shown in Figure 2d, so its water contact angle became the largest one.

#### 3.2.6. Mechanical Properties of PLA/BC Composite Scaffolds 

As a tissue engineering scaffold material, BC has insufficient mechanical properties to provide better support and protect surrounding tissues, while PLA has good mechanical properties as a high polymer. Therefore, we tested the tensile properties of the PLA/BC composites. Statistical comparisons were performed using SPSS 16.0 software (V 16.0, IBM, NewYork, NY, USA) and the difference was considered significant for *p* < 0.05. The results showed that the fracture strength of pure BC was 25.61 MPa (*p* < 0.01). The BC membrane possessed a three-dimensional structure formed by the disordered cross entanglement of fiber bundles composed of extensive nanoscale-fibers. Abundant hydrogen bonds existed in the same molecular chain, endowing the BC membrane with good mechanical strength. After 3D printing, the fracture strength of the composite scaffolds increased up to 59.88 MPa (*p* < 0.02), 66.49 MPa (*p* < 0.01), and 57.81 MPa (*p* < 0.03), respectively. This showed that the composite process of PLA and BC improved its mechanical properties significantly, which proved the strong interaction between BC and PLA, as revealed in the FTIR study. 

### 3.3. Biological Properties Evaluation of PLA/BC Composite Scaffolds

Biocompatibility is the focus of tissue engineering scaffold materials in clinical research, and is one of the important conditions to evaluate the safety of biomaterials. It is necessary to investigate the degradation performance of several scaffolds, whether there is cytotoxicity caused by solvent residue, whether the surface microstructure of composite scaffolds can provide a good environment for the growth of cells or biological tissues, and whether cells can adhere to their surfaces, too evaluate whether the material can promote the growth of cells and materials.

#### 3.3.1. Degradation Properties In Vivo

**Figure 7:**
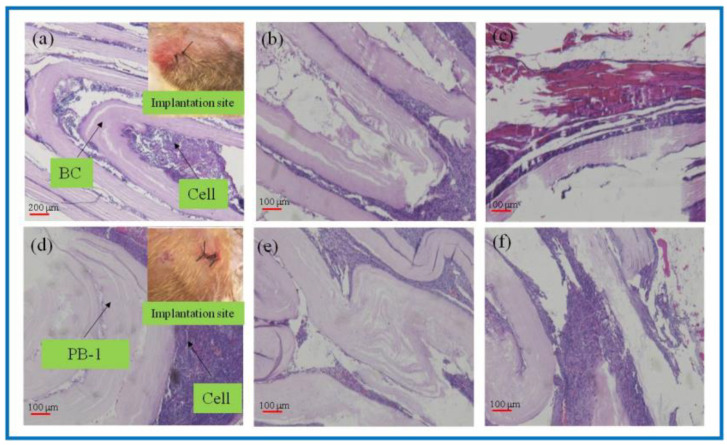
H&E-stained tissue sections embedded in vivo. (**a**–**c**): BC membrane implantation for 1–3 weeks; (**d**–**f**): PLA/BC-1 scaffold implantation for 1–2 weeks.

As a natural material, BC has good biocompatibility and has been widely used in the field of tissue engineering. However, the lack of cellulases in the human body environment, as well as the weak alkaline environment in vivo, makes its degradation process greatly hindered, and studies have shown that, although BC is able to degrade in vivo, the degradation process is slow. PLA could degrade into small molecules of lactic acid in the body environment as a kind of high polymer, while being absorbed by the body, and do not cause toxicity or inflammatory reactions. In this study, in vivo degradation characterization of the BC and 3D-printed PLA/BC-1 composite membrane was performed. The BC membrane and the PLA/BC-1 composite scaffolds were embedded in the inner thighs of New Zealand white rabbits, and data were taken after 1, 2, and 3 weeks, as shown in Figure 7. 

As known from Figure 7a–c, after the BC membrane was embedded in vivo for 1 week, the structure became evacuated and the cells grew into the evacuated interlayer structure. After 2 weeks, the evacuated structure of the BC began to break down, and 3 weeks later, the BC degraded to an increasing extent. It is known from Figure 7d–f that the 3D-printed PLA/BC-1 composite membranes also exhibited a similar degradation state to that of the BC. After 1 week, the structures began to become evacuated and were accompanied by cell ingrowth. After week 2, the layered membranes began to fall off and disperse until week 3, when the degradation phenomenon became obvious and defects in the membrane structure appeared. In summary, the incorporation of PLA accelerated the degradation rate of the composite scaffolds in vivo.

**Figure 8:**
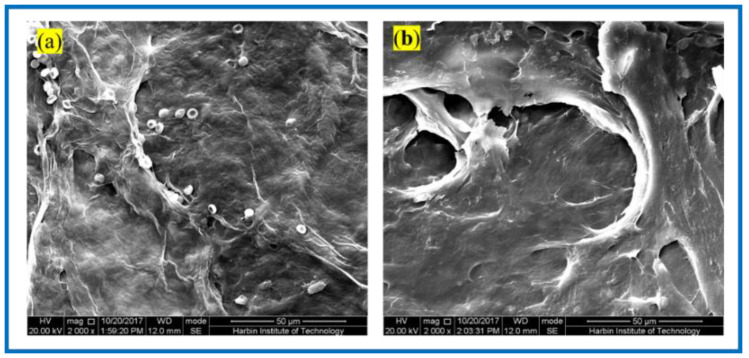
SEM photos taken 3 weeks after composite scaffolds embedded in vivo: (**a**) pristine BC membrane and (**b**) PLA/BC-1 composite scaffold.

Figure 8 shows the SEM of the BC membrane and 3D-printed PLA/BC-1 composite membrane embedded in the inner thighs of New Zealand white rabbits for 3 weeks. After the BC membrane was embedded in white rabbits for 3 weeks, its peripheral tissues adhered and stretched on the surface of the material, grew normally on its surface, and defects appeared on the surface of the BC membrane material, indicating some degradation of the material. Similarly, tissues and cells were also well populated on the surface of 3D-printed PLA/BC-1 composite membranes, which indicated that the materials provided good adhesion sites for the growth of cells, which were able to grow on their surface and migrate towards the inside of the materials, while forming a well-organized structure on their surface. The occurrence of obvious defects in the flat PLA structure on the surface of the material indicated a certain degree of degradation due to the partial physical transition to its highly ordered structure in the complex in vivo environment, as well as during the growth of cells and tissues.

#### 3.3.2. Erythrocyte Fixation Experiments

**Figure 9:**
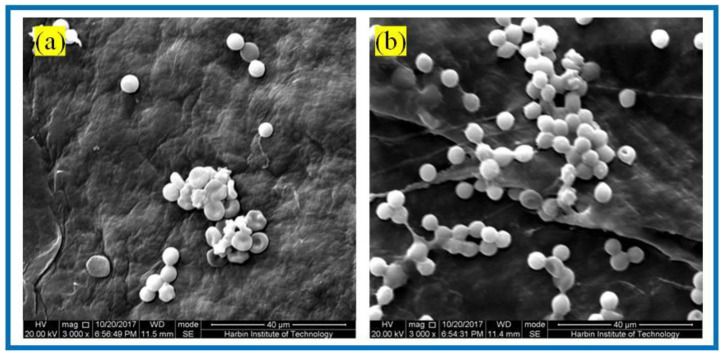
SEM of RBCs fixation of: (**a**) BC membranes and (**b**) PLA/BC-3 composite scaffolds.

For biomaterials applied in vivo, biocompatibility is one of the most major evaluation criteria. Herein, red blood cell fixation experiments and platelet adhesion experiments were used to evaluate the hemocompatibility of tissue engineering scaffolds. In this experiment, rabbit blood cells (RBCs) were used as experimental blood, and anticoagulant was added into the blood to prevent its clotting. RBCs were immobilized on the BC membranes and PLA/BC-3 composite scaffolds, respectively. The adhesion of RBCs was observed by SEM, as shown in Figure 9.

It is known from Figure 9 that abundant RBCs adsorbed on the surface of both the BC membrane and PLA/BC-3 composite scaffolds. Among them, the number of adsorbed RBCs on the surface of the PLA/BC composite membranes was much larger than that on the surface of the BC membranes. RBCs were uniformly adsorbed on the surface of the composite membranes and maintained the intact morphology. This illustrated that the 3D-printed surface microstructure, which increased the specific surface area, provided favorable conditions for the adhesion of RBCs, and was able to promote the adhesion of RBCs on the surface of the membrane layer.

#### 3.3.3. Platelet Adhesion Experiments

**Figure 10:**
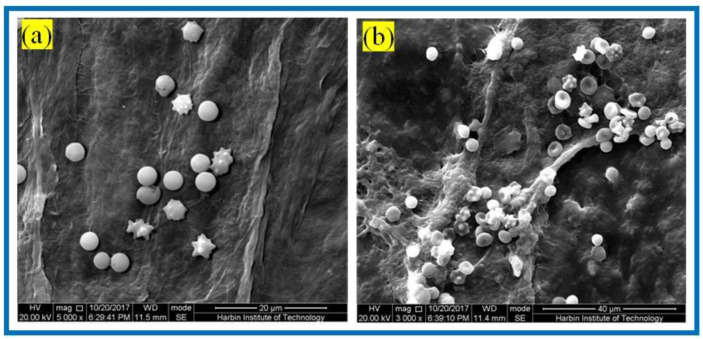
SEM of platelet adhesion on (**a**) BC membranes; (**b**): PLA/BC-3.

Hemostasis is one of the main physiological functions of platelets, and when blood vessels are traumatized, platelets rapidly aggregate and adhere to the bleeding location and form a thrombus to realize hemostasis. Figure 10 shows the SEM of platelet adhesion on BC membranes and PLA/BC-3 composite scaffolds.

It is known from Figure 10 that amounts of platelets adhered to the surface of both the BC membrane and the 3D-printed PLA/BC-3 composite membranes; platelets were well distributed on the surface of the membrane and maintained their intact morphology. The successful adhesion of platelets illustrated that the scaffold material was able to provide adhesion sites for platelets, and therefore, platelets adhering to the surface of the scaffold were able to devote to a certain degree of hemostasis during the process of scaffold embedding.

#### 3.3.4. Cytotoxicity Experiment

Both BC and PLA have good biocompatibility and have been widely used in the field of tissue engineering. The nanoscale three-dimensional network structure on the surface of BC and PLA/BC composite scaffolds provide a large number of adhesion sites for the growth of cells; meanwhile, it is required that the materials do not cause any damage to the surrounding tissues and organs after implantation in vivo. Therefore, performing in vitro toxicity evaluation is a rapid, simple, and highly effective method for biocompatibility evaluation.

In this paper, the direct impregnation method was used to evaluate the sample toxic effect on cell growth. The toxicity of the BC membrane and thes3D-printed PLA/BC composite scaffolds were evaluated respectively. In this experiment, HUVECs were selected to evaluate the toxicity of each of the respective materials. The results are shown in Table 2. Statistical comparisons were performed using SPSS 16.0 software, and the difference was considered significant for *p* < 0.05.

Table 2 shows the relative proliferation rates after HUVEC cells were co-cultured with BC membranes and the three respective 3D-printed PLA/BC composite scaffolds for 48 h. Their cell viabilities were all above 100%, indicating that the BC membrane and the PLA/BC composite membrane can promote the cell proliferation process to some extent. It is known from the toxicity evaluation grading table that HUVEC cells, whether on BC membranes or PLA/BC composite membranes, have a relatively good proliferation rates, with a toxicity grade of 0. 

#### 3.3.5. SCs Adhesion Experiment

In addition to the above direct impregnation method, the direct contact method is also a common biocompatibility evaluation method. The direct contact method was further used to study the adhesion of cells on the sample surface. We selected SCs to evaluate the cytocompatibility of the BC membrane and PLA/BC composite membranes. SCs are a kind of glial cell unique to the peripheral nervous system, which play an important role in nerve regeneration. Therefore, by observing the growth of SCs on the scaffold, we could intuitively evaluate the feasibility of scaffold application in peripheral nerve repair, explore a new way for peripheral nerve tissue repair, and provide experimental reference for the preparation of scaffolding applied in tissue engineering. 

**Figure 11:**
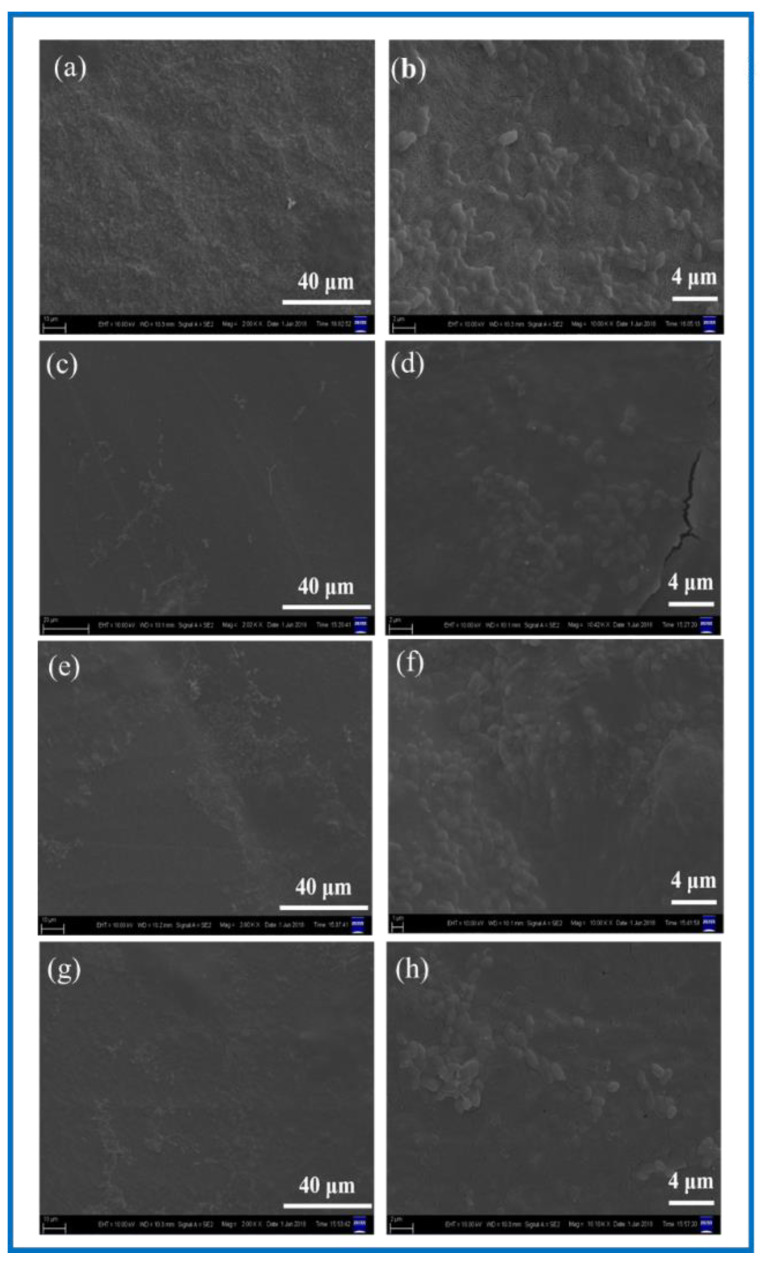
SEM of cell adhesion at different magnification. (**a**,**b**) BC membrane and 3D-printed PLA/BC composite scaffolds: (**c**,**d**) PLA/BC-1; (**e**,**f**) PLA/BC-2; (**g**,**h**) PLA/BC-3.

It is known from Figure 11 that after the 24 h co-culture of the SCs with the BC membranes and PLA/BC composite membranes, cells could successfully adhere on the surface of these several materials. Contrasting the plots by Image J software revealed that the SCs had the highest number of adhered cells on the surface of the BC membrane and the cells were well distributed. There was no obvious reduction in the number of adhered cells on the surface of PLA/BC composite membranes, but agglomerations appeared. This illustrates that by compounding PLA with BC by means of 3D printing, although there was no significant reduction in the number of cell adhesions on its surface, the adhesion pattern was altered, and the cell agglomerations were unevenly distributed.

#### 3.3.6. Analysis of Immunological Staining Results

**Figure 12:**
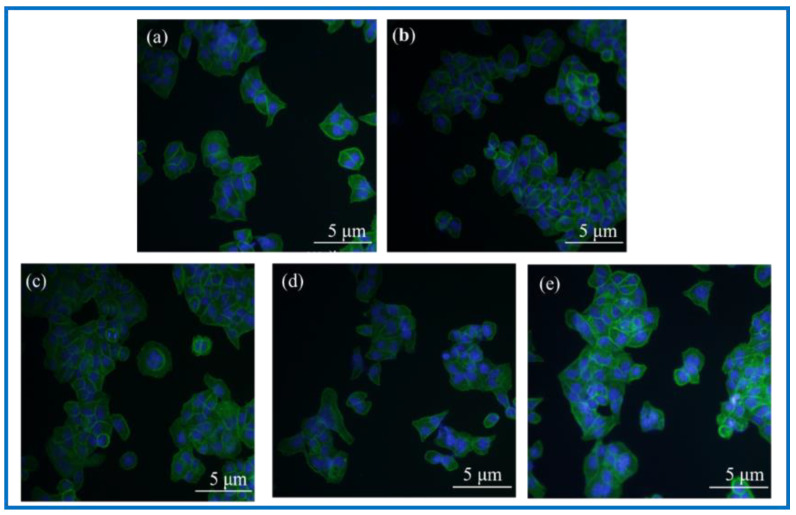
Photographs of fluorescence staining of cell immunology after 24 h co-culture with materials. (**a**): Blank group; (**b**): BC membrane; (**c**): PLA/BC-1; (**d**): PLA/BC-2; (**e**): PLA/BC-3.

DAPI, namely 4’, 6-diamidino-2-phenylindole (4’, 6-diamidino-2-phenylindole), is a fluorescent dye capable of binding to the DNA in cells for cell staining, and is commonly used in fluorescence microscopy observation. DAPI can penetrate the cell membrane to bind to double stranded DNA in living cells to play a labeling role, which produces more than 20 times stronger fluorescence than DAPI itself under a fluorescence microscope, using light of the ultraviolet light wavelength for excitation, which emits blue-green light. Phalloidin, a polypeptide substance isolated from a highly toxic ground mushroom (Amanita phalloides) belonging to the fimbria class of toxins, is a strong toxin, and phalloidin can bind to the F-actin in cells, thus showing their cytoskeletal distribution. To observe the adhesion status of cells on the surface of BC membranes and PLA/BC composite membranes, DAPI staining and FITC-labeled phalloidin staining were performed on the cells and scaffolds composites. Photographs of the fluorescence staining of cell immunology after a 24 h co-culture with the materials are shown as Figure 12.

Observation of Figure 12 shows that the fluorescence blue in the photograph is the nucleus of SCs cells, and the fluorescence green is the cytoskeleton. The three kinds of 3D-printed PLA/BC composite membranes all showed a higher number of cells on the surface than the BC membrane. Linking the cytotoxicity results, the following conclusions can be drawn: (1) the number of adherent cells on the surface of BC membranes and PLA/BC composite membranes increased compared with the blank group, indicating that several materials can provide a good environment for SCs adhesion and growth, and can promote their proliferation processes; (2) The three-dimensional network structure of the pure BC membrane provided adhesion sites for the SCs and was able to promote the cell adhesion process, and the 3D-printed PLA/BC composite membranes exhibited a higher number of adherent cells on the surface, which indicated that the 3D-printed manner resulted in a better environment for cell adhesion and better promoted cell adhesion.

#### 3.3.7. Cell Proliferation Experiments

**Figure 13:**
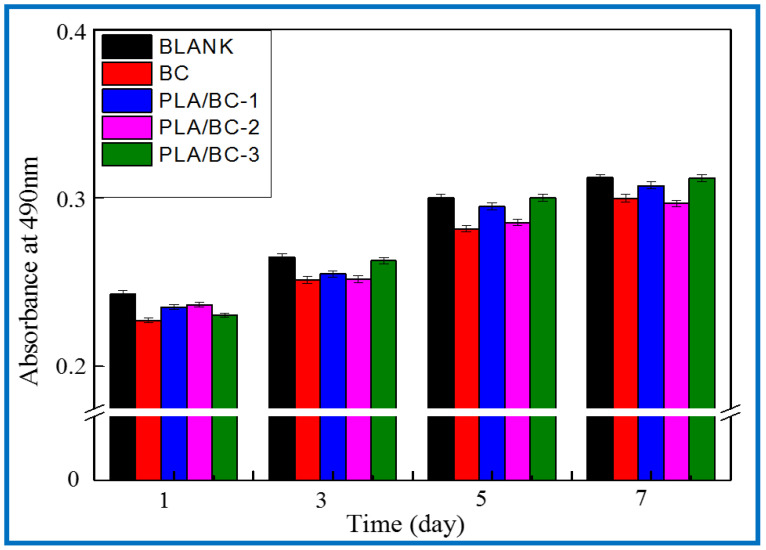
Proliferation of SCs after 1, 3, 5, and 7 d of culturing on BC membranes and PLA/BC composite scaffolds.

After verifying the cytotoxicity of the BC membranes and PLA/BC composite scaffolds, we further investigated the growth of SCs on tissue engineering scaffolds while exploring the effect of scaffolds with different surface microstructures on SCs biological behavior. SCs are the major glial cells of the peripheral nervous system and play a considerable role for growth and development, as well as morphological and functional maintenance of peripheral nerves. Upon nerve injury, SCs are activated from the quiescent phase and secrete a variety of trophic factors that express for nerve regeneration, provide sufficient nutrition for the regeneration process of the nerve, and guide the direction of axonal regeneration by secreting nerve basement membrane components. In this study, the proliferation status of SCs cells cultured on the materials for 1, 3, 5, and 7 d was characterized by MTT assay. Figure 13 shows the proliferation of the SCs cultured on the BC membranes and PLA/BC composite scaffolds for 1, 3, 5, and 7 d. 

It can be seen from Figure 13 that the OD 490 nm value of the BC membrane and the three 3D-printed composite membranes were above 0.22 when the SCs were co-cultured with cells for 1 d, and the gap was not obvious. After cell culture entered 5 d, cell proliferation entered the logarithmic phase, the OD 490 nm value had a significantly higher value, and the gap between groups increased. The OD 490 nm values of the 3D-printed composite membranes were significantly higher than those of the BC membrane group, which indicates that the 3D-printed structures had a certain promoting effect on cell proliferation. At day 7 of cell culture, the proliferation rate of the cells slowed down and the OD 490 nm value changed less, but the 3D-printed PLA/BC composite membranes still showed obvious advantages, with the obvious promotion of proliferation compared with pure BC membranes, which was related to the architecture of their surface microstructures, indicating that the 3D printing process can indeed provide a good environment for the adhesion and proliferation of cells. It is known from Table 3 that during the 7d co-culture of cells and materials, the RGR of the SCs were calculated and were all above 80%, and the material toxicity grade was grade 0 according to the cytotoxicity evaluation criteria as shown in Table 4, which can be used in the field of tissue engineering.

## 4. Conclusions

In this experiment, BC membranes were successfully prepared, and then PLA and BC were combined to prepare new scaffold PLA/BC composite scaffolds by 3D-printed methods. Its physicochemical and biological properties were characterized and evaluated, and the following conclusions were obtained: the 3D printing method not only achieved the composite of the two materials, but also could precisely regulate the micropore size and shape of the PLA layer of the scaffolds by controlling the process parameters during 3D printing, thus exploring the optimal membrane layer structure required for cell adhesion and proliferation. In this paper, we characterized the effects of circular pore-like and stripe-like surface topologies on cell growth behavior. The proliferation rate of the SCs cells on the surfaces of regular round pore-like and stripe-like structures was greater than that on the smooth and flat surfaces, and the micron-sized pore structures could better promote the proliferation process. Meanwhile, the 3D-printed composite membrane had good hemocompatibility and played a role in hemostasis while being applied as a scaffold material for tissue engineering.

## Figures and Tables

**Table 1:** Porosity of BC membrane and PLA/BC composite scaffold.

Group	Porosity (%)
BC	93.75 (*p* < 0.03)
PLA/BC-1	87.85 (*p* < 0.02)
PLA/BC-2	85.58 (*p* < 0.01)
PLA/BC-3	80.95 (*p* < 0.02)

**Table 2:** Cell viability of HUVEC on BC membrane and PLA/BC composite scaffolds.

CV (%) Group	HUVEC
BC	146.78 (*p* < 0.03)
PLA/BC-1	111.71 (*p* < 0.01)
PLA/BC-2	121.48 (*p* < 0.01)
PLA/BC-3	120.92 (*p* < 0.02)

**Table 3:** Relative Growth Rate of BC membranes and PLA/BC composite scaffolds.

CV (%) Days of Culture/d	BC	PLA/BC-1	PLA/BC-2	PLA/BC-3
1	93.52	96.85	97.41	94.81
3	94.85	96.20	95.07	99.17
5	93.88	98.31	95.08	99.99
7	96.06	98.47	95.05	99.86

**Table 4:** Grade of toxicity.

RGR (%)	Grade of Toxicity
80–100	0
60–79	1
40–59	2
20–39	3
0–19	4

## Data Availability

The data that support the findings of this study are available from the corresponding author upon reasonable request.
